# A GdAlO_3_ Perovskite Oxide Electrolyte-Based NO_x_ Solid-State Sensor

**DOI:** 10.1038/srep37795

**Published:** 2016-11-25

**Authors:** Yihong Xiao, Dongmei Wang, Guohui Cai, Yong Zheng, Fulan Zhong

**Affiliations:** 1 National Engineering Research Center of Chemical Fertilizer Catalyst (NERC-CFC), School of Chemical Engineering, Fuzhou University, Gongye Road No.523, Fuzhou 350002, Fujian, P. R. China

## Abstract

NO_*x*_ is a notorious emission from motor vehicles and chemical factories as the precursor of acid rain and photochemical smog. Although zirconia-based NO_*x*_ sensors have been developed and showed high sensitivity and selectivity at a high temperature of above 800 °C, they fail to show good performance, and even don’t work at the typical work temperature window of the automotive engine (<500 °C). It still is a formidable challenge for development of mild-temperature NO_*x*_ detector or sensor. Herein, a novel amperometric solid-state NO_*x*_ sensor was developed using perovskite-type oxide Gd_1−*x*_Ca_*x*_AlO_3−*δ*_(GCA) as the electrolyte and NiO as the sensing electrode. NO_*x*_ sensing properties of the device were investigated at the temperature region of 400–500 °C. The response current value at −300 mV was almost linearly proportional to the NO_*x*_ concentration between 300 and 500 ppm at 500 °C. At such a temperature, the optimal sensor gave the highest NO_2_ sensitivity of 20.15 nA/ppm, and the maximum response current value reached 5.57 μA. Furthermore, a 90% response and 90% recover time to 500 ppm NO_2_ were about 119 and 92 s, respectively. The excellent selectivity and stability towards NO_*x*_ sensing showed the potential application of the sensor in motor vehicles.

The nitrogen monoxide (NO) and nitrogen dioxide (NO_2_), referred as NO_*x*_, are one kind of the most hazardous air pollutants causing acid rain and photochemical smog^1^. A major source of NO_*x*_ emission is from automobile exhaust, and as a result, the NO_*x*_ emission sharply inclined due to the rapid increase of the amount of automobiles worldwide. Therefore, detection and monitoring of NO_*x*_ gas is an important operation in environmental protection. Computerized control of internal combustion engines has improved the work efficiency and decreased the emission of NO_*x*_ gas, where the sensor tracing nitrogen oxide (NO_*x*_) is the key to the closed loop feedback control of the emissions^2^.

Development of NO_*x*_ sensors based on solid electrolytes has attracted great attention recently^3^. The solid electrolytes mainly include two categories: fluorite (AO_2_)-type and perovskite (ABO_3_)-type electrolyte. The traditional solid electrolytes for sensing NO_*x*_ are zirconia-based ceramics with the fluorite (AO_2_)-type structure. Until recently, Yttria stabilized Zirconia (YSZ) based sensors aroused great attention due to their great sensitivity, excellent selectivity, response signal testability, simple structure, the superiority on a wide gas test range, and particularly, the operating ability under high-temperature and hazardous conditions^4^,^5^,^6^,^7^,^8^,^9^,^10^,^11^. Miura *et al*.^12^ fabricated an amperometric-type NO sensor using YSZ substrate with oxide electrode (CdCr_2_O_4_), which showed quick and selective response to NO. Park *et al*.^13^ reported a mixed-potential-type NO_*x*_ sensor using the YSZ electrolyte with a CuO electrode showing good transient responses and large response values. However, the YSZ-based sensors show high oxygen-ion conductivity only above 800 °C. The high operating temperature can lead to a series of problems such as electrode aging^14^, adverse reactions and interfacial diffusion between electrode and electrolyte^15^, weak long term stability and high cost of interconnects^16^ and other issues. This greatly limits the application of such sensors in NO_*x*_ detection. Therefore, it is urgent to search an alternative solid electrolyte material that can work for sensing NO_*x*_ at a mild temperature.

Recently, many mild-temperature NO_*x*_ sensors have been studied. For example, Wang *et al*.^17^ investigated the Pt/La_10_Si_5_NbO_27.5_ (LSNO)/NiO sensor and showed that the sensor had a very high sensitivity to NO_2_ at the operating temperature of 450–600 °C. Dai *et al*.^18^ also fabricated an amperometric-type NO_2_ sensor using Ce_0.9_Gd_0.1_O_1.95_ (CGO) substrated with La_0.75_Sr_0.25_Cr_0.5_Mn_0.5_O_3−δ_ (LSCM) sensing electrode, which gave the high NO_2_ sensitivity of 134 nA/ppm at 500 °C. In addition, Ueda *et al*.^19^ reported that the electrochemical gas sensor Pt/YSZ/La_0.6_Sr_0.4_Co_0.98_Mn_0.02_O_3_, which demonstrated the fast response to NO_2_ at 500–600 °C, but the response current value had only 3 μA to 400 ppm NO_2_ at 550 °C.

At present, perovskite (ABO_3_)-type oxides have been widely studied as potential candidates for gas sensing^20^,^21^,^22^,^23^. Structurally, the AO_2_ type oxides only offer A site for aliovalent ion doping. However, not only the perovskite oxides provide A site but also B site for doping, thus vacancies in the oxygen sublattice are more easily formed and the higher conductivity can be achieved. For example, the oxide ion conductivity exhibiting in the doubly-doped La_0.8_Sr_0.2_Ga_0.83_Mg_0.17_O_2.815_ (LSGM) perovskite oxide is three times higher than 8YSZ at 800 °C^24^,^25^. Sinha *et al*.^26^ showed that calcium-doped GdAlO_3_ is promising material for oxygen-ion-conducting solid electrolyte application. Among the doped systems, Gd_0.85_Ca_0.15_AlO_3−*δ*_ showed a conductivity of 0.057 S/cm at 1000 °C, which was only slightly less than that of yttria-stabilized zirconia at the same temperature. Moreover, the gadolinium aluminate material was widely applied in different luminescent display systems^27^,^28^,^29^,^30^,^31^, neutron absorption, and control rod^32^,^33^,^34^,^35^,^36^. It was also reported that the gadolinium aluminate material could be applied to the solid oxide fuel cell^37^,^38^. However, to the best of our knowledge, no reports were found on NO_*x*_ sensors that are prepared using calcium-doped GdAlO_3_ system as solid electrolyte.

In this paper, an amperometeric NO_*x*_ sensor was fabricated using perovskite-type oxide Gd_1−*x*_Ca_*x*_AlO_3−*δ*_(GCA) as an electrolyte, NiO as sensing electrode (NiO-SE), and a noble metal Pt as reference electrode (Pt-RE), as illustrated in Fig. 1. The GCA powder was synthesized by citrate gel route. The advantage of citrate gel route over the conventional solid-state synthesis method, particularly for singly and doubly doped GdAlO_3_ compositions, is that it tunes at a molecular level and produces solid powders that could be sintered to have good densities at significantly lower temperatures^39^. NO_2_ sensing performance of the device was measured on the Electrochemical Workstation to study its sensitivity, selectivity and stability at the temperature range of 400–500 °C.

## Results and Discussion

### Characterization of the sensor materials

**Figure 2:**
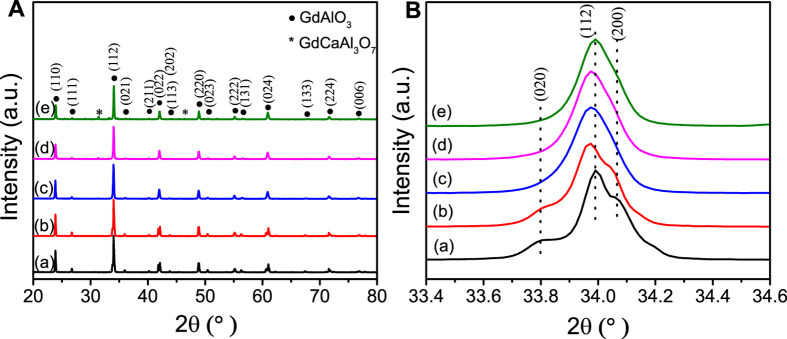
(**A**) XRD patterns of Gd_1−*x*_Ca_*x*_AlO_3−*δ*_powders calcined at 1500 °C for 4 h: (a) x = 0, (b) x = 0.05, (c) x = 0.1, (d) x = 0.15, (e) x = 0.2, and (**B**) enlarged portion at 2*θ* = 33.6–34.4°.

XRD patterns and an expanded view around 2*θ* (33.6–34.4°) of the samples calcined at 1500 °C for 4 h are shown in Fig. 2A and B, respectively. As shown in Fig. 2A, when *x* was in the range of 0–0.1, the diffraction peaks of the resulting samples were indexed to orthorhombic crystal structure of GdAlO_3_ phase [ICDD PDF 46–0395]. However, for *x* = 0.15 and 0.2, additional diffraction peaks were observed in the XRD patterns, indicating the emergence of a impurity phase indexed to the tetragonal crystal structure of GdCaAl_3_O_7_ [ICDD PDF 50–1808]. It showed that the Ca doping is limited in the GdAlO_3_ lattice structure and less than 15%. The result is highly consistent with the literature^26^. For *x* = 0.05–0.15, it was noticed from Fig. 2B that the corresponding Bragg diffraction, 2*θ*, shifted towards lower values and the doublet (112, 200) was merged into a single peak.

We used the following Eq. 1 for further analyzing the measured XRD patterns. Based on the {110}, {112}, and {024} peaks, the lattice parameters for a given symmetry can be calculated as shown in Fig. S1. The Ca doping at *x* = 0.05 does not change *a* value, whereas *b* decreases significantly from 8.49 Å to 5.95 Å and *c* increases from 6.47 Å to 7.47 Å. With increasing the Ca^2+^ concentration to 15%, these lattice parameters don’t further change, indicating that the Ca^2+^ doping only resulted in the *d*_*hkl*_ expansion. Clearly, the 2*θ* shift to lower values was resulted from the lattice expansion. It was attributed to the difference in ionic size because the Ca^2+^ (0.134 nm) ion was bigger than the Gd^3+^ (0.127 nm) for coordination number 12^26^.


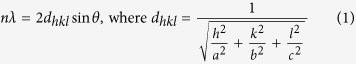


Here, *n* is the diffraction order, *λ* is the x-ray wavelength, *d*_*hkl*_ is the interplanar distance, (*h k l*) are the Miller indices for the corresponding *d*-spacing and *a*, *b*, *c* are the lattice parameters.

Additionally, it can be observed that with increasing *x*, the three peaks of {020, 112, 200} are gradually merged into one peak and decreased in intensity. The gradual broadening and weakening are mainly originated from the lattice disordering. For *x* = 0.2, the {112} Bragg diffraction peak is nearly coincided with *x* = 0.0. It was attributed to the phase change of GdAlO_3_ doped with Ca^2+^ ion, leading to the formation of a new matter, GdCaAl_3_O_7_, due to the dissociation of doped Ca^2+^ ions from the GdAlO_3_ lattice structure. The most different point is that the 2*θ* position of the {112} peak recovers for x = 0.2, but the broadening and weakening of these diffraction peaks are reserved, suggesting that the local structural disordering is maintained by the formation of oxygen vacancies.

**Figure 3:**
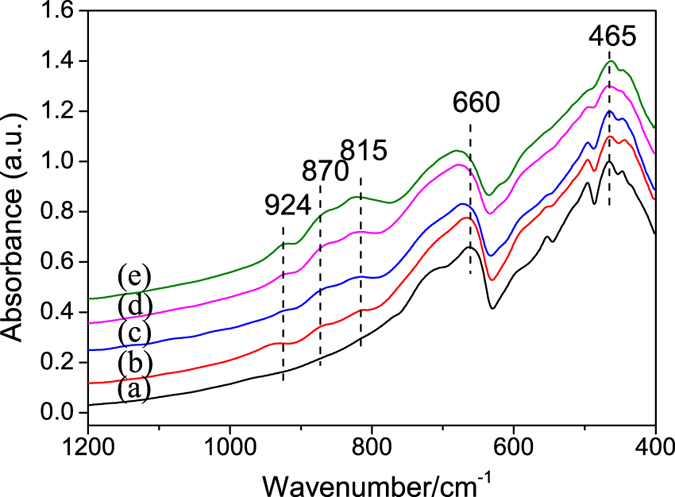
Infrared spectra of Gd_1−*x*_Ca_*x*_AlO_3−*δ*_powders prepared by calcinations of gel precursors for 4 h at 1500 °C: (**a**) x = 0, (**b**) x = 0.05, (**c**) x = 0.1, (d) x = 0.15, and (**e**) x = 0.2.

Figure 3 shows the IR spectra of the samples prepared by calcinations of gel precursors for 4 h at 1500 °C. It was observed that the spectra measured at different Ca-doped concentrations are basically the same except for a systematic data shift. The IR spectra showed strong bands at 660 and 465 cm^−1^ which are characteristic M-O (possibly Gd-O and Al-O stretching frequencies) vibrations for the perovskite structure compounds^40^. Additionally, it was noted that the Ca-doped samples showed three new peaks at 815, 870, and 924 cm^−1^ and the peaks at 660 cm^−1^ moved to a higher wave number with increasing *x*. It has been reported that the size-induced lattice variations and the concentration of oxygen vacancies might lead to a red shift of IR absorption^41^,^42^. For the Gd_1−*x*_Ca_*x*_AlO_3−*δ*_systems, since the diameter size of Ca^2+^ ion is bigger than that of Gd^3+^ ion, it results in the lattice expansion and formation of oxygen vacancies. The Ca-doped GdAlO_3_ point defect reaction could be written as Eq. 2^26^:


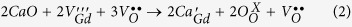


Here, with increasing *x*, the concentration of mobile oxygen vacancies increased. Moreover, these new IR peaks are originated from the formation of some new chemical bonds by introduction of calcium. The above results suggested that Ca^2+^ ions have entered into the perovskite lattice structure.

**Figure 4:**
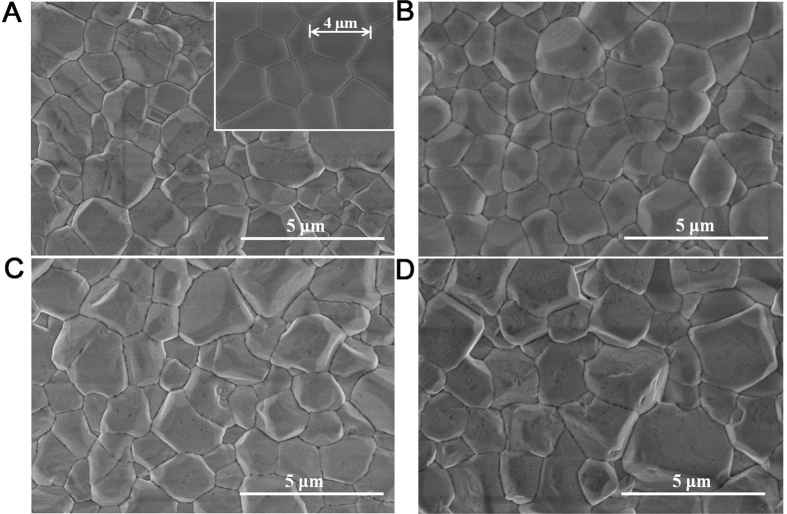
SEM images of the surfaces of Gd_1−*x*_Ca_*x*_AlO_3−*δ*_substrates calcined at 1500 °C for 4 h (**A**) x = 0.05, the inset shows the x = 0, (**B**) x = 0.1, (**C**) x = 0.15, and (**D**) x = 0.2.

Figure 4 shows the SEM images of surface of the Gd_1−*x*_Ca_*x*_AlO_3−*δ*_substrate calcined at 1500 °C for 4 h. The surface was consisted of microparticles with sizes between 1 and 5 μm, and no open pore could be seen. The surface morphology of the undoped gadolinium aluminate sample is shown in inset of Fig. 4A. Compared to the undoped sample, it can be seen that there is no significant change in morphology and surface structure for Gd_1−*x*_Ca_*x*_AlO_3−*δ*_substrates. Only the average grain sizes increased slightly with the increase of Ca doping concentration.

### Sensing performance of the devices

This sensor based on the solid electrolyte substrates can be shown as the following electrochemical cells in the presence of O_2_ and NO_2_: (−) O_2_ + NO_2_, NiO/GCA/Pt, NO_2_ + O_2_ (+). When the sensor was exposed to the sample gas, the response current value changed at the fixed potential of −300 mV. The following electrochemical reactions would occur at the triple-phase boundary (TPB, among gas/sensing electrode/electrolyte) and counter electrode in a series of physisorption and charge exchange reactions, as described by Eq. 3 and Eq. 4.


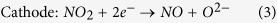



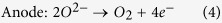


Amperometric response and recovery transients to 500 ppm NO_2_ with a polarized potential of −300 mV at 400, 450, and 500 °C for the sensor based on Gd_0.85_Ca_0.15_AlO_3−*δ*_substrate were shown in Fig. S2. As well known, if you choose the cathode to be positive in the software setup, and a negative voltage of −300 mV is applied in the work, you will obtain a positive current. As seen from Fig. S2, the baseline current increased with increasing temperature due to some reactions of low level impurities at electrode and slight electric conductive contribution, which was in agreement with the result reported by Wang *et al*.^43^.

**Figure 5:**
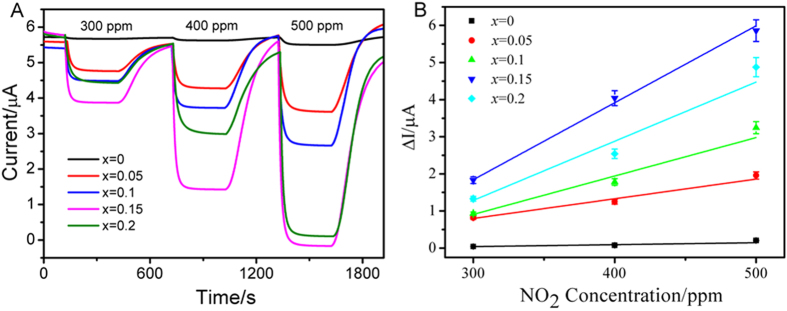
(**A**) Response transients of the sensor based on Gd_1-*x*_Ca_*x*_AlO_3−*δ*_ substrates to 300–500 ppm NO_2_ at 500 °C in the presence of 5 vol% O_2_ (applied potential −300 mV, flow rate 200 cm^3^/min); (**B**) The relationship between the response current values and NO_2_ concentrations.

Figure 5A shows the response transients of the sensor based on Gd_1−*x*_Ca_*x*_AlO_3−*δ*_ substrates at 500 °C under various NO_2_ concentrations in the range between 300 to 500 ppm in the presence of 5 vol. % O_2_, when the potential of −300 mV was applied. In order to better distinguish the response curve, the base current levels have been shifted. At each NO_2_ concentration, a large increase in response current value was observed in the case of the sensor based on Gd_1−*x*_Ca_*x*_AlO_3−*δ*_substrate. For instance, the response current value of the sensor based on Gd_0.9_Ca_0.1_AlO_3−*δ*_substrate was 0.93 μA for 300 ppm NO_2_ at 500 °C. When the NO_2_ concentration went up to 400 ppm, the response current value inclined to 1.78 μA. For the NO_2_ sensor in this study, the NiO sensing electrode showed a strong adsorption and catalytic activity for NO_2_. An increase of the NO_2_ concentration brought enhancement of NO_2_ adsorption on the sensing electrode, which would produce more oxygen ions (O^2−^) through the cathodic reaction of Eq. 3 and thus the response current of the sensors was improved.

In this work, the response current value was defined as the difference of current value between the sample gas and base gas (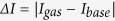, where *I*_*gas*_ and *I*_*base*_ referred to the current values in the targeted concentration *C* ppm and 0 ppm NO_2_). Figure 5B shows the relationship between the response current of the sensor based on Gd_1−*x*_Ca_*x*_AlO_3−*δ*_substrate and the NO_2_ concentration at a bias potential of −300 mV. It can be seen that the response current value was almost linear to the NO_2_ concentration from 300 to 500 ppm for the sensor based on Gd_1−*x*_Ca_*x*_AlO_3−*δ*_substrate at 500 °C. The sensitivity of the sensor is defined as the ratio of response current to NO_2_ concentration. For *x* = 0.05–0.15, the sensitivity of the sensor was enhanced with increasing the Ca doping. For example, the sensitivities of the sensors based on Gd_1−*x*_Ca_*x*_AlO_3−*δ*_substrate with *x* = 0.05, 0.1 and 0.15 were 5.71, 11.58 and 20.15 nA/ppm at 500 °C, respectively. Nevertheless, the sensitivity of the sensor based on the Gd_0.8_Ca_0.2_AlO_3−*δ*_ substrate declined to 19.22 nA/ppm because of over-doping. Compared to the response transient of the reference sensor based on YSZ-8 substrate to 500 ppm NO_2_ in the presence of 5 vol% O_2_ at 500 °C (see Supplementary Fig. S3), the sensor based on Gd_1−*x*_Ca_*x*_AlO_3−*δ*_ substrates showed the potential application in motor vehicles.

**Figure 6:**
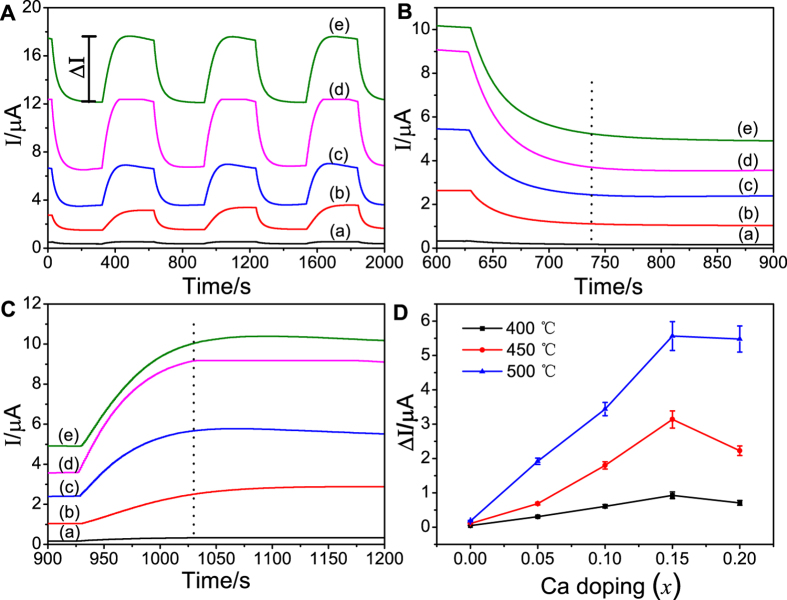
(**A**) Response transients of the sensor based on Gd_1−*x*_Ca_*x*_AlO_3−*δ*_ substrates to 500 ppm NO_2_ in the presence of 5 vol% O_2_ at 500 °C (applied potential −300 mV, flow rate 200 cm^3^/min): (a) x = 0, (b) x = 0.05, (c) x = 0.1, (d) x = 0.15, (e) x = 0.2; (**B**) and (**C**) showed the enlarged portions of (**A**) response transients in the range between 600 to 1200 s; (**D**) the effect of calcium doping on the response current value of gadolinium aluminate system in 500 ppm NO_2_ at 400–500 °C.

Figure 6A shows the response transients of the sensor to 500 ppm NO_2_ at 500 °C and a bias potential of −300 mV. In order to better distinguish the response curve, the base current levels have been also shifted. It was observed that the response current values increased steadily from the base level upon switching from the base gas to the sample gas. The current quickly recovered to the original level when the sensor was exposed to the base gas. The response and recovery times are important parameters used to characterize a sensor. The response time is defined as the time that the resistance of the sensor reaches to 90% of the saturation value when the sensor was exposed to NO_2_ and the recovery time is defined as the time required for recovering 90% of the original resistance^44^. The response transients in the ranges of 600–900 and 900–1200 s are shown in Fig. 6B and C, respectively. The response and recovery time initially cut down along with the Ca doping. The response time for *x* = 0.05, 0.1, and 0.15 was 136, 121 and 119 s at 500 °C, respectively. For *x* = 0.2, the response time was extended to 178 s at 500 °C. Clearly, the sensor based on Gd_0.85_Ca_0.15_AlO_3−*δ*_substrate is the optimal device for sensing NO_2_ owing to the shortest response (119 s) and recovery (92 s) time.

Figure 6D shows the effect of calcium doping on the response current values (ΔI) of the sensor based on the gadolinium aluminate system substrates at different temperatures. The current values of the undoped sample were 0.049 μA at 400 °C. With increase of Ca doping, ΔI reached 0.31, 0.61 and 0.93 μA for *x* = 0.05, 0.1, and 0.15 at 400 °C, respectively. However, ΔI of *x* = 0.2 dropped to 0.7 μA, suggesting that ΔI greatly increased by doping Ca. As seen from Fig. 6D, ΔI was enhanced with increasing the operating temperature. The sensor based on Gd_0.85_Ca_0.15_AlO_3−*δ*_substrate exhibited the highest ΔI value of 5.57 μA at 500 °C. The response currents of the Ca-doped GdAlO_3_ sensors were one order magnitude higher than that of the undoped GdAlO_3_. The increase in current value may be attributed to the increased conductivity of the Ca-doped GdAlO_3_ substrates. For the perovskite-type oxides, tolerance factor *t*, can be used for describing the relationship between symmetry and ionic radii (Eq. 5):


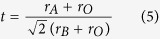


Here, *r*_*A*_ is the ionic radii of Gd^3+^, *r*_*B*_ is the ionic radii of Al^3+^, and *r*_*O*_ is the ionic radii of O^2−^. Theoretically, when *t* is equal to 1, the orthorhombic structure of perovskite-typed GdAlO_3_ will be altered to a cubic structure with a higher symmetry. The Ca doping made *t* approach to 1, as result of the larger ionic radius of Ca^2+^ (0.134 nm) than that of Gd^3+^ (0.127 nm) of GdAlO_3_ for coordination number 12^26^, consequently enhancing the conductivity of GdAlO_3_. Furthermore, by the Ca doping, the cell volume of the perovskite will increase and the concentration of mobile oxygen vacancies that assist the mobility of oxygen ion will increase. In brief, for *x* = 0.05–0.15, the Ca doping GdAlO_3_ solid electrolyte is favorable for conductive properties of the sensor, whereas for *x* = 0.2, the conductivity begins to decrease due to the formation of impurity phase.

**Figure 7:**
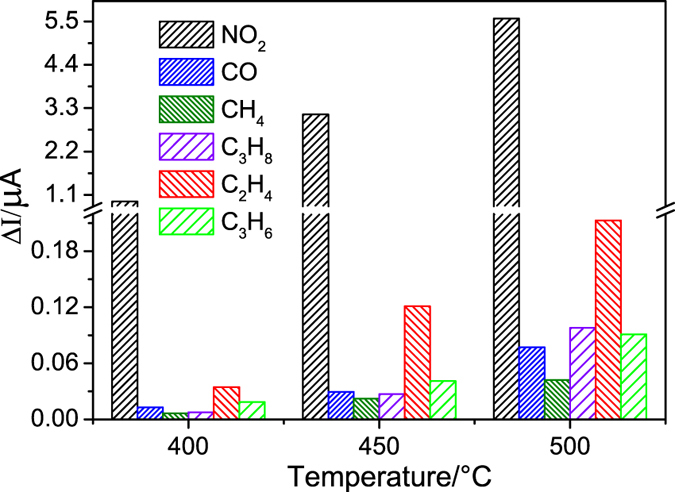
Selectivity of the sensor based on Gd_0.85_Ca_0.15_AlO_3−*δ*_ substrate in 500 ppm various gases at 400, 450 and 500 °C, respectively (applied potential −300 mV, flow rate 200 cm^3^/min).

The selectivity is defined as the response discrepancy of an indicated gas from a mixed gas. Here, the selectivity factor is defined as S = I_A_/I_B_, where I_A_ and I_B_ are the responses of a sensor to a target gas A and an interference gas B, respectively^45^. To evaluate the selectivity, the responses of the sensor based on Gd_0.85_Ca_0.15_AlO_3−*δ*_substrate to CO, CH_4_, C_3_H_8_, C_2_H_4_ and C_3_H_6_ were examined. Figure 7 shows the responses of different gases with a concentration of 500 ppm. It was observed that the sensor exhibited a high sensitivity and selectivity towards NO_2_ gas compared to the other gases. The ΔI value of interference gases was fairly small in comparison with that of the sensor responding to NO_2_ gas. For example, the ΔI values of NO_2_, CO, CH_4_, C_3_H_8_, C_2_H_4_ and C_3_H_6_ were 5.57, 7.7 × 10^−2^, 4.20 × 10^−2^, 9.80 × 10^−2^, 2.13 × 10^−1^, and 8.97 × 10^−2^ μA at 500 °C, respectively. And the selectivity factors to CO, CH_4_, C_3_H_8_, C_2_H_4_ and C_3_H_6_ were 72.34, 132.62, 56.84, 26.15 and 62.10, respectively. The influence of O_2_ flow on the selectivity of sensor was negligible (see Supplementary Fig. S4).

**Figure 8:**
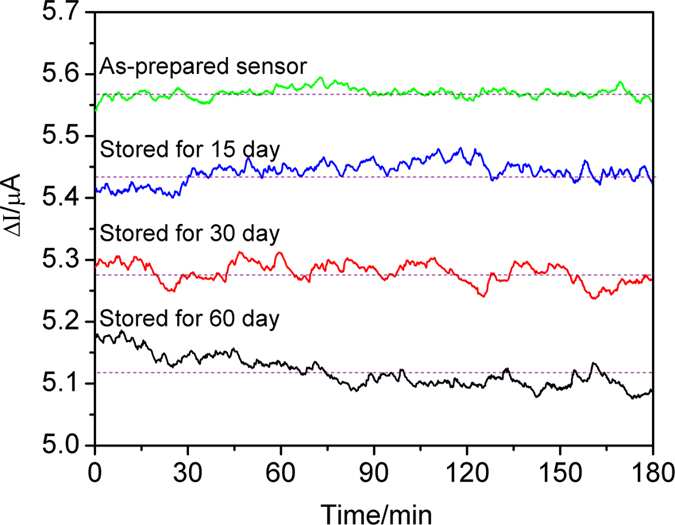
Stability test for the sensor based on Gd_0.85_Ca_0.15_AlO_3−*δ*_ substrate at 500 °C in the presence of 500 ppm NO_2_ (applied potential −300 mV, flow rate 200 cm^3^/min).

The stability of the sensor based on Gd_0.85_Ca_0.15_AlO_3−*δ*_ substrate was measured for 3 h upon exposure to 500 ppm NO_2_ gas with 5 vol. % O_2_ at 450 °C (Fig. 8). The horizontal shows the centerline of the response current ripple. The as-prepared sensor shows a response current (ΔI) of 5.57 μA. Furthermore, after the sensor was stored for half a month, a month, and two months, the response signal decreased slightly by 2.5%, 5.0% and 7.3%, respectively. The response current decreased by about 0.43 μA after the sensor was stored in ambient atmosphere for two months, compared to the as-prepared sensor. These results suggested that the sensor had a good stability.

## Conclusions

In summary, an amperometric sensor based on Gd_1−*x*_Ca_*x*_AlO_3−*δ*_ substrates was developed. It showed excellent sensitivity to NO_*x*_ gas at mild temperatures. The response current values of the sensor enhanced with increase of operating temperature. Furthermore, the sensitivity and response current values initially increased and then decreased with increasing the Ca doping. The Gd_0.85_Ca_0.15_AlO_3−*δ*_ sensor gave the highest NO_2_ sensitivity of 20.15 nA/ppm and the highest response current value of 5.57 μA at 500 °C. Moreover, the sensor also exhibited a great selectivity and excellent stability.

## Methods

### Synthesis and analysis of GCA electrolytes

The Ca-doped GdAlO_3_ powder was prepared through a citrate gel route^26^,^39^. Gd(NO_3_)_3_·6H_2_O (99.99% purity), Al(NO_3_)_3_·9H_2_O (AR Grade), and Ca(NO_3_)_2_·4H_2_O (AR Grade) were used as starting materials for preparation of Gd_1−*x*_Ca_*x*_AlO_3−*δ*_(GCA) samples, where *x* = 0, 0.05, 0.1, 0.15, and 0.2, respectively. The starting materials were taken with composition (Gd_1−*x*_Ca_*x*_):Al = 1:1 molar ratio. For all the compositions, the molar ratio of total metal ion to citrate ion was kept 1. The gadolinium nitrate, aluminium nitrate and calcium nitrate were first dissolved in 250 mL distilled water at room temperature. Then citric acid as complex agent was added to the reaction solutions. Further, the mixed solution was slowly evaporated at 80 °C under stirring to form white transparent gel. The gel was further dried at 120 °C in an oven for 10 h to form precursor powder. After that, the powder was calcined at 1000 °C for 4 h.

The XRD data were first recorded on a Panalytical X’Pert Pro diffractometer at 40 kV and 40 mA using Co Kα_1_ (λ = 0.178 901 nm), then revised by Cu Kα_1_ (λ = 0.154 056 nm). The samples were scanned over a 2θ range from 10° to 100° with a step size of 0.0167° at a scanning rate of 10°·min^−1^. The infrared spectra in the range of 1200–400 cm^−1^ were recorded on a Thermo Fisher Scientific Nicolet 6700 FTIR device. The samples were prepared as KBr pellets.

### Sensor fabrication and characterization

A NO_2_ sensor was fabricated using NiO as sensing electrode (NiO-SE), which was synthesized by using the sol-gel method, perovskite-type oxide Gd_1−*x*_Ca_*x*_AlO_3−*δ*_as electrolyte, and a noble metal Pt as reference electrode (Pt-RE). Pellets with 8 mm in diameter and 3 mm thickness were produced by applying 150 Mpa pressure to the calcined powders, and then sintered at 1500 °C for 4 h in the air. The NiO-SE was made from NiO paste, and a Pt wire (0.2 mm diameter) was attached to the NiO layer as a current collector. In addition, the Pt-RE was formed by using platinum paste, which was painted onto polished surface of GCA pellet which was on the other side, and then a Pt wire (0.2 mm diameter) was attached to the Pt surfaces. Subsequently, the sample was fired at 1000 °C for 1 h in air to get the NiO/GCA/Pt sensor.

Scanning electron microscopy (SEM, HitachiS4800 instrument) was applied for observing the morphology of the samples.

### Evaluation of sensing properties

The fabricated sensor was assembled in a quartz tube and the sensing properties were evaluated in a conventional gas-flow apparatus equipped with a furnace operating at temperatures in range of 400–500 °C (see Supplementary Fig. S5). The gas environment consisted of a changing concentration of NO_2_ (0–500 ppm) with base gases (O_2_ + N_2_ balance) at a total flow rate of 200 cm^3^/min.

The amperometric responses of the sensors were carried out using a potentiostatic method at −300 mV. The fore-mentioned electrochemical measurements were carried out by the Electrochemical Workstation (Instrument corporation of Shanghai, China, CHI600E).

## Additional Information

**How to cite this article**: Xiao, Y. *et al*. A GdAlO_3_ Perovskite Oxide Electrolyte-Based NO_x_ Solid-State Sensor. *Sci. Rep.* **6**, 37795; doi: 10.1038/srep37795 (2016).

**Publisher's note:** Springer Nature remains neutral with regard to jurisdictional claims in published maps and institutional affiliations.

## Supplementary Material

Supplementary Information

## Figures and Tables

**Figure 1:**
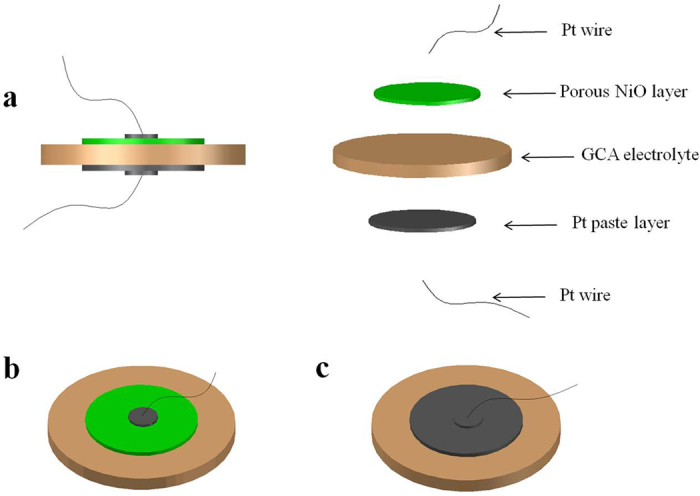
(**a**) Schematic representation of the fabricated sensor, (**b**) top view of the sensor, (**c)** bottom view of the sensor.
