# Supplementary material for: A GdAlO3 Perovskite Oxide Electrolyte-Based NOx Solid-State Sensor
Source: Sci Rep. 2016 Nov 25;6:37795. doi: 10.1038/srep37795 (PMC5122901; doi:10.1038/srep37795)
Supplement: Supplementary Information [file srep37795-s1.pdf]

Supplementary Information

A $GdAlO_3$ Perovskite Oxide Electrolyte-Based NO_x Solid-State Sensor

Yihong Xiao, Dongmei Wang, Guohui Cai, Yong Zheng & Fulan Zhong*

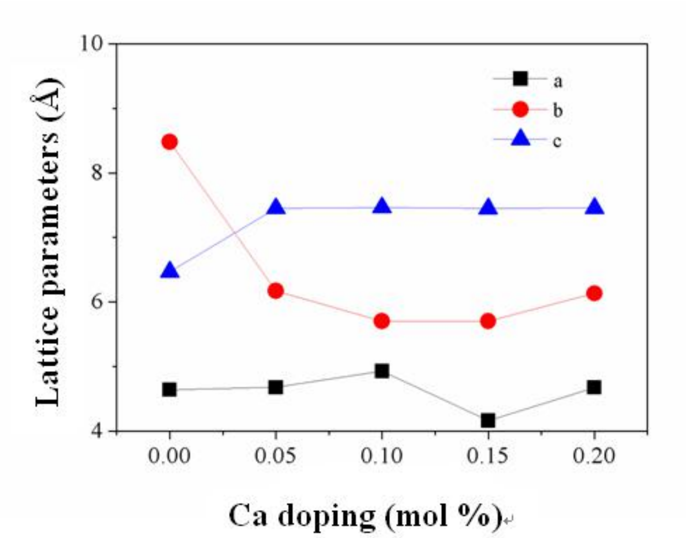

Fig S1.The change in lattice parameters of $GaAlO_3$ with Ca ion doping

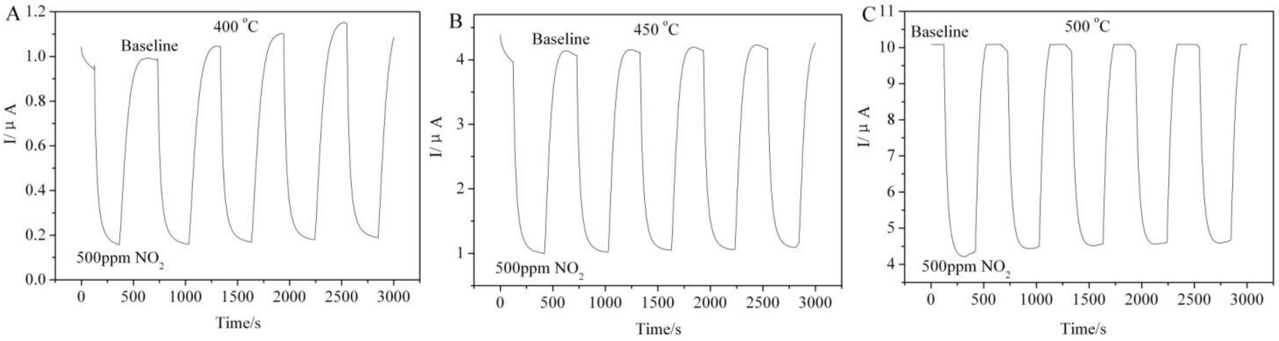

Fig.S2 Amperometric response and recovery transients to 500 ppm NO_2 for the sensor based on $Gd_{0.85}Ca_{0.15}AlO_{3-\delta}$ substrate at 400°C, 450°C, and 500°C (applied potential –300 mV, flow rate 200 cm^3/min).

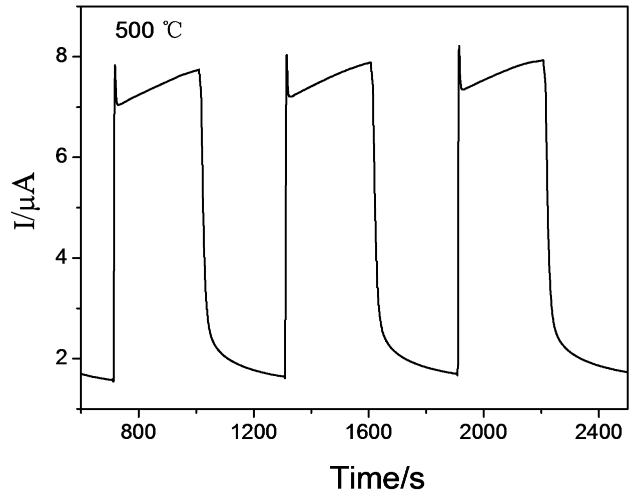

Fig S3. Response transients of the sensor based on YSZ-8 substrates to 500 ppm NO_2 in the presence of 5 vol% O_2 at 500 °C (applied potential –300 mV, flow rate 200 cm^3/min)

Fig. S3 showed the response transients of the sensor based on YSZ-8 substrates to 500 ppm NO_2 in the presence of 5 vol% O_2 at 500 °C. As seen from Fig. S3, the response current value (△I) is about 5.7 μA for the sensor based on YSZ-8 substrates. Under the same conditions, the response current value (△I ≈ 5.57 μA) of Ca-doped $GdAlO_3$ material is as good as commercial YSZ. The sensor based on $Gd_{1-x}Ca_xAlO_{3-\delta}$ substrates showed the potential application in motor vehicles.

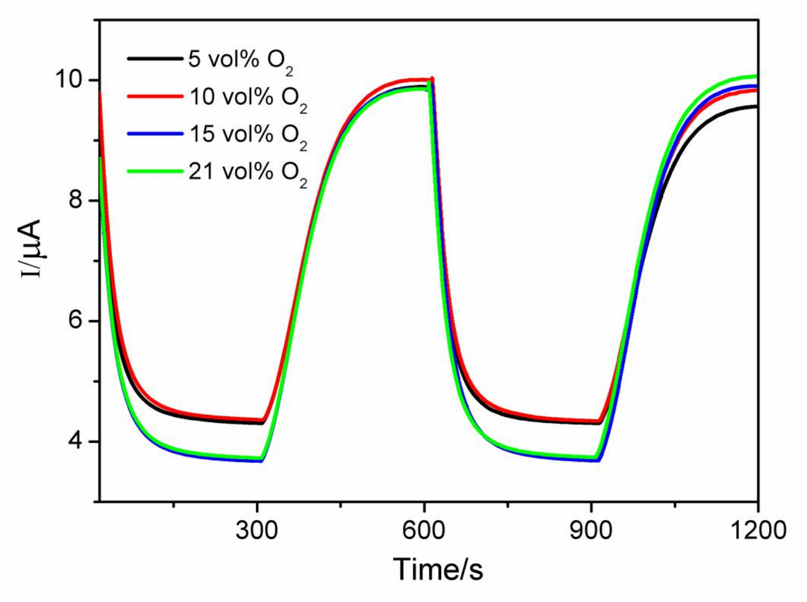

Fig S4**.** Dependence of response transients to 500 ppm NO_2 on concentrations of O_2 (5-21 vol%) for the sensor based on $Gd_{0.85}Ca_{0.15}AlO_{3-\delta}$ substrate attached with NiO-SE at 500 °C (applied potential –300 mV, flow rate 200 cm^3/min).

Fig. S4 showed the dependence of response transients to 500 ppm NO_2 on concentrations of O_2 (5-21 vol%) for the sensor based on $Gd_{0.85}Ca_{0.15}AlO_{3-\delta}$ substrate attached with NiO-SE at 500 °C. It is clear that the response current values in the 500 ppm NO_2 increased slightly with the increase of the O_2 concentration. The maximum change of $\triangle$I was 0.75 μA when the concentrations of O_2 from 10 vol% to 20 vol%. This change is negligible.

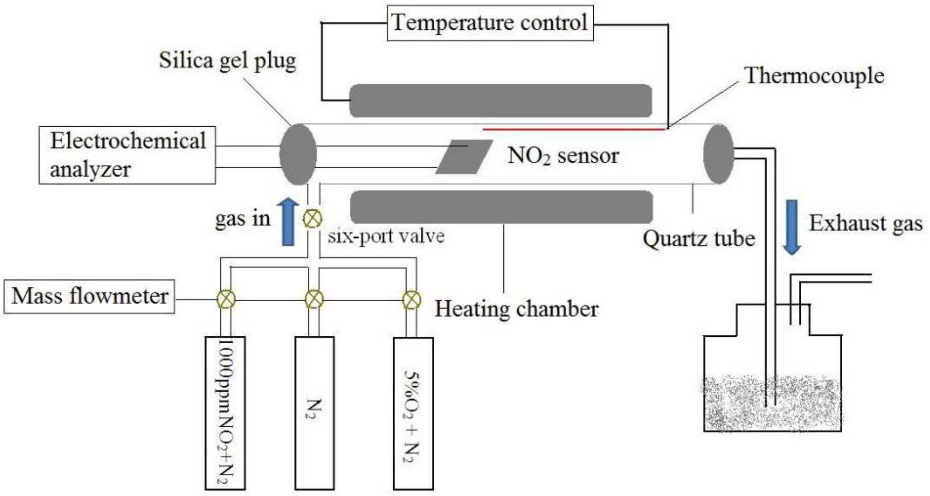

Fig. S5. Experimental setup used for evaluation of NO_2 sensor

The mixed gas is monitored by a six-port valve before reaching the sample, as showed in Fig.S5. Therefore, the switching between NO_2 and supplementary N_2 leads to the delayed response to NO_2, resulting in the increase of the response time.
